# Quantitative and Dynamic MRI Measures of Peripheral Vascular Function

**DOI:** 10.3389/fphys.2020.00120

**Published:** 2020-02-28

**Authors:** Erin K. Englund, Michael C. Langham

**Affiliations:** ^1^Department of Orthopaedic Surgery, University of California, San Diego, La Jolla, CA, United States; ^2^Department of Radiology, University of Pennsylvania, Philadelphia, PA, United States

**Keywords:** MRI, reactive hyperemia, blood flow, endothelial (dys)function, flow mediated dilatation, perfusion

## Abstract

The endothelium regulates and mediates vascular homeostasis, allowing for dynamic changes of blood flow in response to mechanical and chemical stimuli. Endothelial dysfunction underlies many diseases and is purported to be the earliest pathologic change in the progression of atherosclerotic disease. Peripheral vascular function can be interrogated by measuring the response kinetics following induced ischemia or exercise. In the presence of endothelial dysfunction, there is a blunting and delay of the hyperemic response, which can be measured non-invasively using a variety of quantitative magnetic resonance imaging (MRI) methods. In this review, we summarize recent developments in non-contrast, proton MRI for dynamic quantification of blood flow and oxygenation. Methodologic description is provided for: blood oxygenation-level dependent (BOLD) signal that reflect combined effect of blood flow and capillary bed oxygen content; arterial spin labeling (ASL) for quantification of regional perfusion; phase contrast (PC) to quantify arterial flow waveforms and macrovascular blood flow velocity and rate; high-resolution MRI for luminal flow-mediated dilation; and dynamic MR oximetry to quantify oxygen saturation. Overall, results suggest that these dynamic and quantitative MRI methods can detect endothelial dysfunction both in the presence of overt cardiovascular disease (such as in patients with peripheral artery disease), as well as in sub-clinical settings (i.e., in chronic smokers, non-smokers exposed to e-cigarette aerosol, and as a function of age). Thus far, these tools have been relegated to the realm of research, used as biomarkers of disease progression and therapeutic response. With proper validation, MRI-measures of vascular function may ultimately be used to complement the standard clinical workup, providing additional insight into the optimal treatment strategy and evaluation of treatment efficacy.

## Introduction

Blood flow is necessary to sustain life through the delivery of substrates for cellular metabolism including oxygen and nutrients, and the removal of waste products. Regulation of blood flow to tissue is a complex and dynamically controlled process mediated in large part by the vascular endothelium (Furchgott and Zawadzki, 1980). Endothelial dysfunction, the phenotypic presentation of a vasoconstricted, pro-inflammatory, thrombogenic state, underlies many diseases including atherosclerosis (Harrison et al., 1987) and diabetes (Yamauchi et al., 1990), and is present in patients with significant risk factors for cardiovascular disease including smoking (Messner and Bernhard, 2014), aging (Lakatta and Levy, 2003) and hypertension (Zeiher et al., 1993). A reduced bioavailability or activity of nitric oxide is thought to be the predominant mechanism underlying endothelial dysfunction, resulting in reduced vasodilation and delayed vascular reactivity (Davignon, 2004). Other articles in this special issue will focus on the physiologic importance of mediators that maintain vascular homeostasis in the microvasculature and endothelium, but here, we briefly overview some emerging non-invasive magnetic resonance imaging (MRI) methods to evaluate peripheral vascular function in the context of injury and inflammation.

**FIGURE 1:**
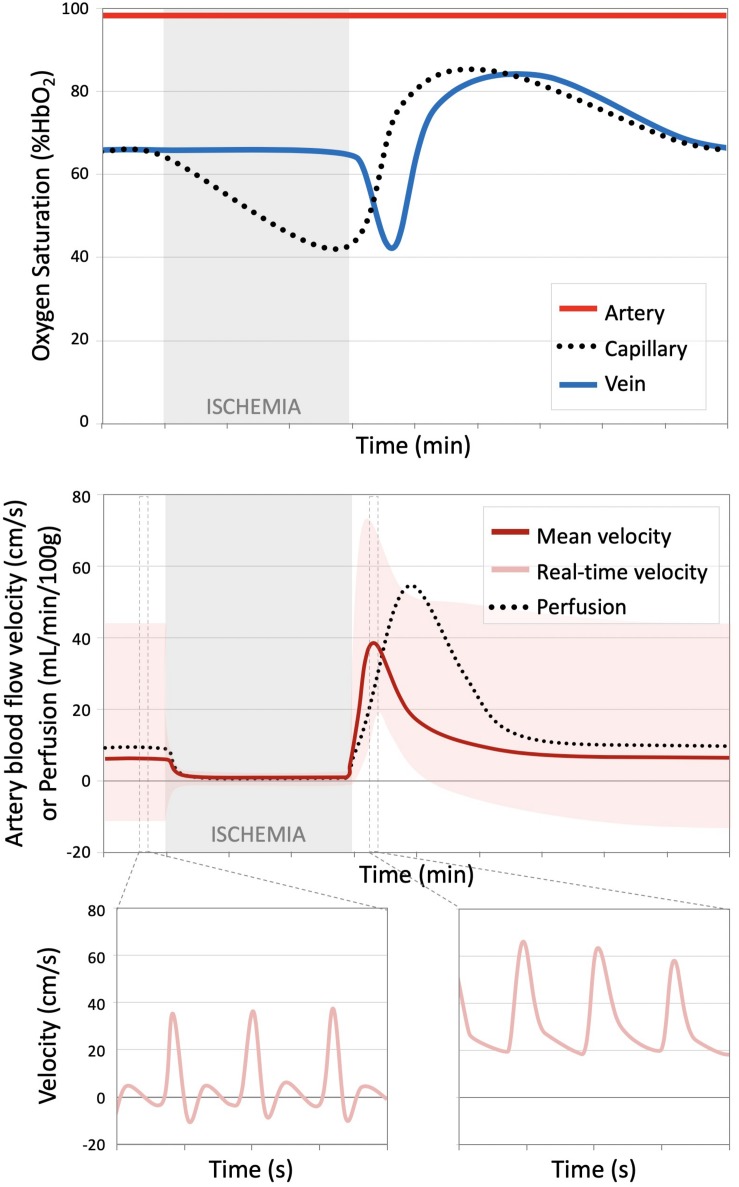
Time course oxygen saturation and blood flow over a reactive hyperemia protocol. (Top) Illustration of hemoglobin oxygen saturation (%HbO_2_) measured in the artery, proximal to the cuff (red), capillary (black dotted line), and venous (blue) circulations. During the period of ischemia, arterial and venous %HbO_2_ remain constant, but progressively decreases in the stagnant blood in the capillaries. Upon cuff release, the deoxygenated blood from the capillaries serves as an endogenous tracer and can be tracked as it flows into the large draining veins. The capillary bed and venous oxygen saturations surpass the baseline condition during hyperemia. (Bottom) Blood flow velocity and tissue perfusion decrease from the relative low baseline value to approximately zero during induced ischemia. Following cuff release, there is a transient surge in arterial flow velocity, which translates to increased perfusion, albeit at a slight lag. This figure shows the mean arterial velocity averaged over a cardiac cycle (dark red) and the real-time flow waveform (light red). The arterial flow waveform, initially triphasic at rest in healthy subjects, becomes entirely antegrade during the period of hyperemia. Dynamic, temporally resolved MRI methods can quantify various aspects of the illustrated processes. Figure adapted from Englund et al. (2013) and Englund et al. (2016) with permission.

In general, assessment of endothelial function can be accomplished by measuring the magnitude and temporal dynamics of blood flow and oxygenation in response to a vasoactive stimulus such as exercise, induced ischemia, or chemical stimulation (e.g., Acetylcholine). To evaluate peripheral vascular function, a reactive hyperemia protocol is commonly used, in which the response following a period of induced ischemia is interrogated (Figure 1). During the period of arterial occlusion, blood flow in the arteries, capillaries, and veins is suspended. The stagnant blood in the capillary bed is subjected to continued oxygen extraction (in short, the desaturated blood serves as an endogenous tracer), though the oxygen diffusion gradient between blood and tissue decreases as a function of ischemic duration (Lebon et al., 1998). Meanwhile, there is local accumulation of vasodilators, activation of inwardly rectifying potassium channels and Na + /K + -ATPase (Crecelius et al., 2013), and a reduction in arteriolar pressure, causing an overall decrease in vascular resistance (Carlsson et al., 1987). Following cuff release, reactive hyperemia ensues with a transient surge of macrovascular flow rate as much as five-fold increase owing to the decrease in microvascular resistance downstream at the level of the arterioles. This increase of blood flow also amplify shear stress at the vessel wall, ultimately triggering additional arteriolar vasodilation (Tagawa et al., 1994; Widlansky et al., 2003). The return of blood flow causes an increase in perfusion, delivering oxygenated blood to the ischemic tissue and driving out the accumulated vasodilators and deoxygenated capillary blood. In the presence of endothelial dysfunction, the reactive hyperemia response in dampened and/or delayed (Fronek et al., 1973; Lieberman et al., 1996; Ledermann et al., 2006; Isbell et al., 2007). Changes in vascular reactivity may therefore provide insight into early, sub-clinical disease states (Flammer et al., 2012).

Conventional physiologic measurements used to assess surrogate markers of endothelial function include ultrasound to quantify blood flow and arterial diameter, strain gauge plethysmography to measure tissue perfusion, and invasive catheterization for blood gas analysis to determine oxygen saturation in the arteries and veins. Though commonly used clinically for evaluation of anatomy, MRI technology is far richer and has the greater potential to quantify a spectrum of physiologic parameters of interest, non-invasively across multiple vascular beds in a single session. Other imaging modalities including contrast-enhanced MRI (Isbell et al., 2007; Zhang et al., 2019), positron emission tomography (PET) (Heinonen et al., 2010), near infrared spectroscopy (NIRS) (Nioka et al., 2006; Baker et al., 2017), and ultrasound (Celermajer et al., 1992) have also been used to evaluate peripheral vascular function. However, non-contrast MRI has the advantage of being spatially resolved (unlike plethysmography) with a fixed frame of reference (compared to NIRS or ultrasound) and is entirely non-invasive (compared to contrast-enhanced MRI or catheterization) and does not expose the subjects to ionizing radiation (in contrast to PET). Specific tailoring of the MRI pulse sequence allows for quantification of various parameters which can be expressed in physiologic units, including for instance, blood flow velocity (cm/s) or flow rate (mL/min), tissue perfusion (mL/min/100g), and oxygen saturation (% hemoglobin oxygen saturation, %HbO_2_).

Here, we review recent developments and results from quantitative, dynamic, non-contrast MRI studies for evaluation of vascular function and reactivity including blood oxygen level-dependent (BOLD) imaging, arterial spin labeling (ASL), phase contrast (PC), including MR-measured pulse wave velocity (PWV), luminal flow mediated dilation (FMD), and dynamic MR oximetry, summarized in Table 1. The goal of this manuscript is to introduce the reader to these MRI methods and to review studies that have employed these methods as biomarkers of disease presence, severity, and in the evaluation of treatment response.

**TABLE 1:** Summary of MRI methods to dynamically evaluate vascular function.

MR method	Measurement	Units	Site	Typical temporal resolution
BOLD	Relative capillary bed oxygen content	% change from baseline	Microvasculature	1–2 s
ASL	Tissue perfusion	mL/min/100g	Microvasculature	2–16 s
PC	Blood flow velocity, flow rate, PWV	cm/s or mL/min	Large arteries, veins	5–60 ms
FMD	Vessel diameter or area	% change from baseline	Large arteries	12 s
Oximetry	Hemoglobin oxygen saturation	%HbO_2_	Microvasculature or large draining veins	2–228 s

*BOLD, blood oxygen level-dependent; ASL, arterial spin labeling; PC, phase contrast; PWV, pulse wave velocity; FMD, flow-mediated dilation.*

## Bold MRI to Assess Capillary Oxygen Content

The most widely used MRI method to evaluate changes in blood flow and oxygenation is the measurement of the BOLD response (Raichle, 1998; Kim and Ogawa, 2012). BOLD MRI is perhaps best known as the basis for functional neuroimaging experiments, providing insight into patterns of neural activity in response to a specified task (Glover, 2011). Similar principles may be applied to the peripheral circulation, allowing BOLD MRI of skeletal muscle to inform on vasoactive changes in response to exercise or induced ischemia.

The BOLD signal arises due to changes in the local content of paramagnetic deoxyhemoglobin. In the context of a reactive hyperemia paradigm, during the period of induced ischemia constant oxidative metabolism in nearby cells will cause blood in the capillary bed to desaturate, increasing the local concentration of deoxyhemoglobin. This accumulation of paramagnetic deoxyhemoglobin will cause the local magnetic field to become more inhomogeneous, ultimately leading to faster decay of the MR signal (e.g., decreased effective transverse relaxation time, T_2_^∗^). Following release of the cuff, the surge of oxygenated blood decreases the concentration of deoxyhemoglobin in the capillaries (increasing T_2_^∗^), while at the same time, expanding the blood volume, which tends to decrease T_2_^∗^. Thus, the BOLD signal serves as a surrogate marker of capillary bed oxygen content, mediated by both changes in blood flow/volume and oxygen extraction. Complicating the physiologic interpretation even more, BOLD signal is also sensitive to changes in cellular pH, vessel diameter, and vessel orientation (Lebon et al., 1998; Damon et al., 2007; Sanchez et al., 2010; Partovi et al., 2012a), and is significantly impacted by static magnetic field inhomogeneities.

Despite the complicated origin of the BOLD signal, its implementation is quite straightforward. Gradient-recalled echo MR images are acquired with echo time approximately equal to the expected T_2_^∗^ (yielding T_2_^∗^- or BOLD-weighted images), or with multiple echoes to quantify T_2_^∗^ from the rate of signal decay. The dynamic data, generally measured at a temporal resolution of 1 s (Huegli et al., 2008; Schulte et al., 2008; Kos et al., 2009; Towse et al., 2016; Tonson et al., 2017; Larsen et al., 2019), are normalized by the baseline signal intensity and the relative changes in response to the vasoactive stimulus (e.g., exercise or induced ischemia) are then evaluated. The relative magnitude of the response (e.g., maximum signal change following cuff release/exercise, or minimum during induced ischemia) and the temporal response kinetics (e.g., time to half maximum/minimum, time to peak response) provide insight into the combined blood flow and oxygenation responses that are occurring locally at the level of the capillary bed.

Early manifestations of endothelial dysfunction, even in the absence of overt cardiovascular disease, have been observed with BOLD MRI. In general, those with risk factors for cardiovascular disease such as older subjects versus younger individuals (Schulte et al., 2008; Kos et al., 2009) and smokers compared to non-smokers (Nishii et al., 2015), demonstrated a blunting and delay of the BOLD response following induced ischemia (i.e., reactive hyperemia). In addition, impaired vascular function has been observed from BOLD imaging in clinically overt diseases including peripheral artery disease (PAD) (Ledermann et al., 2006; Englund et al., 2015; Li et al., 2016; Bakermans et al., 2019), critical limb ischemia (CLI) (Huegli et al., 2008), and systemic sclerosis (Partovi et al., 2012b, 2013). Assessment of treatment response following percutaneous transluminal angioplasty in patients with CLI (Huegli et al., 2008; Bajwa et al., 2016), showed that patients had improvements in the reactive hyperemia response as assessed by BOLD imaging. Another recent study used BOLD MRI to investigate the therapeutic effect of antioxidants on the response to exercise and induced ischemia following eccentric exercise, but contrary to their hypothesis found no significant effect (Larsen et al., 2019).

These results are, in general, promising for the use of BOLD imaging during reactive hyperemia as a biomarker for disease progression and therapeutic response, particularly due to the ease of implementation. However, the BOLD response is inherently measured relative to some initial condition, is not quantified in physiologic units, and it is difficult to separate the contributions of flow and oxygenation changes to the measured signal.

## ASL MRI for Perfusion Quantification

To quantify tissue perfusion in physiologically relevant units, another type of non-contrast MRI acquisition, ASL, can be used (Detre et al., 1992; Williams et al., 1992; Kim, 1995; Kim et al., 1997; Wong et al., 1997). In ASL, a magnetic label (i.e., inversion pulse) is applied to water protons in arterial blood, allowing the blood, serving as an endogenous tracer, to be tracked as it flows from the large arteries into the capillary bed and perfuses the tissue. The perfusion signal is isolated by pairwise subtraction between two images, the second acquired without application of the arterial tagging pulse. The difference between these two images removes the contribution from static background tissue, leaving behind only signal related to perfusion of the labeled blood. This signal can be converted into perfusion in physiologic units of mL/min/100g through application of various models that describe the exchange of the labeled protons between arterial blood and tissue (Buxton et al., 1998; Raynaud et al., 2001; Alsop et al., 2014).

In reactive hyperemia experiments, the time course of perfusion, measured at temporal resolution up to 2 s (e.g., Englund et al., 2016) is analyzed to determine the speed (e.g., time to peak), and magnitude (e.g., peak hyperemic flow) of the post-ischemic perfusion response. Unlike BOLD, the magnitude of the response quantifies the amount of tissue perfusion, and the timing is unimpacted by the concurrent changes in capillary bed oxygen saturation. Prior studies have uncovered an association between these ASL-based measures and PAD disease presence and severity (Wu et al., 2009; Englund et al., 2015). Compared to healthy controls, patients with PAD had a decrease in peak perfusion, and with worsening disease severity, there was a prolongation of the time to peak perfusion.

In addition to the muscles of the leg, perfusion of the foot has also been interrogated (Zheng et al., 2014), finding that patients with diabetes had impaired perfusion following toe extension exercise compared to healthy controls. Finally, investigation of the impact of percutaneous transluminal angioplasty with reactive hyperemia ASL measurements showed that patients with CLI had improvements in perfusion in some but not all muscles following intervention (Grözinger et al., 2013), which may be reflective of the heterogeneity seen between muscles prior to intervention (Wu et al., 2008, 2009).

While ASL measures microvascular perfusion, the blood flow response in the capillary bed following induced ischemia is mediated by both the macro- and microvascular reactivity. This means that it’s not possible to separate the effects from macrovascular stenoses from potential primary microvascular dysfunction. In PAD, the macrovascular lesions are generally unmodified by conservative therapies such as exercise (Sanne and Sivertsson, 1968), thus there is significant interest in isolating the microvascular response. Efforts to disentangle these two effects has come in the form of physiologic models of the reactive hyperemia perfusion response (Chen and Wright, 2017), or through simultaneous measurement of microvascular and macrovascular blood flow responses (Englund et al., 2017). Additional work to clearly define and isolate the microvascular contribution is necessary.

## Phase Contrast MRI for Quantification of Macrovascular Flow

In addition to quantifying perfusion in the microcirculation, MRI can also be used to measure blood flow in the large arteries and veins via PC. In PC-MRI, magnetic field gradients are used to encode the motion of the water protons into the signal phase (Moran, 1982; Bryant et al., 1984; Moran et al., 1985). A thorough technical explanation of PC-MRI can be found in Nayak et al. (2015), but briefly, application of bi-polar magnetic field gradients will impart a residual phase offset in moving protons relative to static tissue as a function of velocity and gradient parameters (e.g., duration, temporal separation, and strength). Since the timing and gradient strength are chosen pulse sequence parameters, the measured phase can be converted to velocity (in cm/s).

By gating the phase contrast acquisition to the electrocardiogram, time-resolved images of blood flow throughout the cardiac cycle can be reconstructed (Nayler et al., 1986). Acquisition generally takes several seconds to minutes depending on the desired apparent temporal resolution, as data are sampled over several heartbeats and synthesized together to create images over the cardiac cycle. In young healthy subjects, the baseline flow waveform of arteries in the high-resistance peripheral circulation (e.g., superficial femoral artery) is triphasic, with high velocity antegrade flow during the systolic phase of the cardiac cycle, followed by retrograde flow during early and late diastole, respectively. In contrast, patients with flow-limiting stenoses in the peripheral arteries generally have a monophasic flow waveform, attributed to decreased vascular resistance distal to the stenosis (Akbari, 2012). In these patients, the flow profile loses the retrograde flow during diastole, remaining antegrade throughout the cardiac cycle (Bernstein et al., 1970; Mohajer et al., 2006; Langham et al., 2013a; Versluis et al., 2014).

Furthering this technique, velocity vector components can be resolved by encoding motion in each of the three directions in succession, and if 3-dimensional spatial encoding is implemented at the same time, 4D flow images can be reconstructed (i.e., velocity vector is time-resolved (Buonocore, 1998; Frydrychowicz et al., 2007). 4D flow MRI data allow assessment of complex flow dynamics and can provide striking visualization of the impact of macrovascular lesions, showing flow jets, vortices, and areas of turbulence, which may be useful for understanding the patterns of atherosclerotic plaque development (Markl et al., 2012). Additionally, simultaneous quantification of the temporally resolved blood flow waveform at two distinct locations along the artery of interest can be used to compute PWV, a measure of arterial stiffness (Wentland et al., 2014). In contrast to the traditional arterial applanation tonometry, MR-measured PWV is not limited to superficial arteries, can provide accurate path length measurements, and can probe shorter segments of the artery, providing regional PWV of the aortic arch or femoral artery (Langham et al., 2011).

In addition to this baseline characterization of the arterial flow waveform, the dynamics of the arterial blood flow response during reactive hyperemia (Mohiaddin et al., 2002; Langham et al., 2010b, 2013a, 2015) or following exercise (Englund et al., 2017) provide insight into endothelial function, vascular reactivity, and flow reserve (Versluis et al., 2012). By quantifying macrovascular blood flow dynamically in the feeding artery, the time to peak flow and the duration of forward flow can be measured. These parameters are increased in the presence of PAD (Langham et al., 2013a) and are sensitive to early changes in endothelial function that occur with smoking, aging (Langham et al., 2015), and have recently been shown to be acutely impaired following nicotine-free e-cigarette aerosol inhalation (Caporale et al., 2019).

## Flow-Mediated Dilation for Evaluation of Endothelial Function

Perhaps the most widely studied surrogate marker of endothelial function is ultrasound measurements of FMD in the brachial artery following cuff-induced ischemia (Celermajer et al., 1992). While often regarded as an effective surrogate marker for endothelial function (or dysfunction), the method’s poor intra-subject reproducibility (Hardie et al., 1997) plague brachial artery FMD. The reported coefficients of variation of FMD measurements vary widely from as little as 1.5% to approximately 50% in others (Sorensen et al., 1995; Andrews et al., 1997; Hardie et al., 1997; de Roos et al., 2001). This limitation is magnified since the average magnitude of FMD is approximately 5% (Boushel and Piantadosi, 2000) and ultrasound settings such as dynamic range, gain and probe distance are known to significantly affect diameter measurements (Potter et al., 2008). For these reasons, the value of ultrasound-based FMD measurement in routine clinical practice has been put into question (Bhagat et al., 1997; Pyke and Tschakovsky, 2005; Sejda et al., 2005) since its introduction over 25 years ago.

Magnetic resonance imaging (MRI) can also be used to quantify vessel cross-sectional area through a variety of measures including phase contrast angiography (Silber et al., 2001), high-resolution cine bright-blood imaging (Wiesmann et al., 2004), and dynamic vessel wall imaging methods (Langham et al., 2013b). In addition to the measurement of vessel diameter needed for FMD quantification, these MRI methods, depending on the sequence used, can be used to evaluate the vessel wall or flow dynamics. Brachial artery FMD measured by MRI was found to be lower in smokers compared to non-smokers (Wiesmann et al., 2004), and in long-term users of birth control (contraceptive depot medroxyprogesterone acetate) during menstruation compared to control women with no intake of progestogens (Sorensen et al., 2002).

A new approach to rapidly acquire high-resolution vessel-wall images to assess plaque burden in PAD have been modified to quantify superficial femoral artery FMD at 60, 90, and 120 s after cuff release (Langham et al., 2013b, 2016). Of note is that the luminal FMD (denoted *FMD*_*L*_), consisting of a measurement of the change in cross-sectional area (*FMD*_*L*_≡δ*A*/*A*_*o*_≈2δ*r*/*r*_*o*_), where δ*r* and r_0_ are the changes in radius, and radius at rest, respectively, yields greater detection sensitivity compared to ultrasound-based-measurement of the change in arterial diameter, i.e., *FMD*≡δ*d*/*d*_0_ = δ*r*/*r*_*o*_. Recent work indicates that the superficial femoral artery *FMD*_*L*_ is sensitive enough to detect acute effects of nicotine-free electronic cigarette aerosol inhalation (Caporale et al., 2019).

## Dynamic Oximetry for Quantification of Vascular Reactivity

While the previous methods generally focused on the dynamic quantification of blood flow, measurement of blood oxygen saturation (e.g., %HbO_2_) may also be useful for understanding the underlying tissue metabolism. Differences in the magnetic susceptibility of oxygenated and deoxygenated hemoglobin (Pauling and Coryell, 1936) can be exploited to quantify %HbO_2_ in the microvasculature based on the irreversible transverse relaxation time, T_2_’ (He and Yablonskiy, 2006), or %HbO_2_ in large vessels via T_2_- (Lu and Ge, 2008) or MR susceptometry- (Haacke et al., 1997; Fernández-Seara et al., 2006) based oximetry. Furthermore, when these measures of oxygen extraction are combined with the previously described measures of blood flow, the muscle oxidative metabolism can be computed via Fick’s principle (Zheng et al., 2013; Mathewson et al., 2014; Englund et al., 2017).

In addition, temporally resolved measurement of intravascular venous oxygen saturation (SvO_2_) throughout an ischemia-reperfusion paradigm allows for the intravascular blood to act as an endogenous tracer as it transits from the capillary bed to the large draining vein. During the period of induced ischemia, oxygen extraction continues in the stationary blood of the capillary bed and upon cuff release, the hyperemic arterial inflow drives deoxygenated blood from the capillary bed into the collecting veins, causing the measured SvO_2_ to drop sharply (Langham et al., 2010a, 2013a, 2015; Langham and Wehrli, 2011). Thus blood flow, tissue metabolism, and endothelium-mediated dilation underlie the measured SvO_2_ dynamics measured in the large draining vein.

Washout time (time to minimum SvO_2_), upslope – representing the rate of resaturation (maximum slope during recovery), and overshoot (peak SvO_2_ minus baseline SvO_2_) can be extracted from the SvO_2_ time course data. These metrics reflect the reactivity of the microvessels to NO-mediated vasodilation. Langham et al. revealed an association between alterations in the SvO_2_ time course-derived metrics in the femoral (Langham et al., 2010a; Langham and Wehrli, 2011) or posterior tibial (Englund et al., 2015) veins and the presence of PAD. Compared to age-matched healthy controls and young healthy subjects, patients with PAD had a longer washout time, diminished upslope, and lower overshoot, suggesting endothelial dysfunction. Furthermore, these dynamic measurement of SvO_2_ are altered in pre-clinical disease states including aging and smoking (Langham et al., 2015), and most recently have been shown to be sensitive to acute effects of nicotine-free e-cig aerosol inhalation (Caporale et al., 2019).

## Discussion

The methods and results described herein illustrate the vast capability of MRI for dynamic evaluation of endothelial function. While there are many other approaches to quantify blood flow, arterial diameter, or oxygen saturation, MR imaging is the only modality capable of providing all parameters, non-invasively, without being limited by depth or radiation exposure. However, many of the described methods are not standard acquisition schemes available on clinical MRI scanners. Thus, there is a need for open-access to the acquisition and image analysis software packages, which would help to expand the availability of these advanced methods to researchers without dedicated MR physicists and image analysis experts.

In general, the findings reviewed herein revealed that the reactive hyperemia response was blunted and delayed in various diseases and conditions with underlying endothelial dysfunction, regardless of the measurement method, corroborating prior non-MR-based research (e.g., Fronek et al., 1973). While these methods have been described in the context of investigation of peripheral vascular function, similar strategies could be used to measure cerebrovascular reactivity albeit in response to different vasoactive stimuli (Fisher et al., 2018).

Use of MRI and selection of imaging contrast may ultimately help to unveil the mechanism of action for disease progression or therapeutic response. For example, it is known that exercise improves pain-limited walking distance in patients with PAD (Murphy et al., 2012), but the mechanism is not entirely understood. Using MRI, changes in tissue perfusion could be used to assess the contribution of microvascular angiogenesis, while measurement of venous oxygen saturation may provide insight into changes in the mitochondrial efficiency and metabolic processes, and the combined effect of these factors may be unveiled by BOLD imaging. Finally, MR-measured perfusion, FMD, and oximetry could replace plethysmography, ultrasound, or invasive catheter-based measures for studies investigating the specific signaling pathways involved in vasodilation (e.g., Crecelius et al., 2013), or the effect of dietary supplements on blood flow during exercise (e.g., Richards et al., 2018).

The methods described herein have thus far been largely relegated to the realm of research. Use as a clinical tool and biomarker for disease progression and therapeutic response mandates that the accuracy, precision, and repeatability of the measurements be well documented, and that the methods be accessible on clinical scanners. Future studies combining such MRI methods with clinical measures and outcomes will help to define the additive benefit of these imaging metrics in cohorts of subjects as well as individual patients.

## Author Contributions

EE and ML drafted, edited, and approved the final version of the manuscript.

## Conflict of Interest

The authors declare that the research was conducted in the absence of any commercial or financial relationships that could be construed as a potential conflict of interest. The handling Editor declared a shared affiliation and past co-authorship with one of the authors ML.

## References

[B1] Akbari C. M. (2012). “Clinical features and diagnosis of peripheral arterial disease,” in *The Diabetic Foot*, eds Piaggesi A. Apelqvist J. (Totowa, NJ: Humana Press), 75–85. 10.1007/978-1-61779-791-0_5

[B2] Alsop D. C. Detre J. A. Golay X. Günther M. Hendrikse J. Hernandez-Garcia L. (2014). Recommended implementation of arterial spin-labeled perfusion MRI for clinical applications: a consensus of the ISMRM perfusion study group and the European consortium for ASL in dementia. *Magn. Reson. Med.* 73 102–116. 10.1002/mrm.25197 24715426 PMC4190138

[B3] Andrews T. C. Whitney E. J. Green G. Kalenian R. Personius B. E. (1997). Effect of gemfibrozil +/- niacin +/- cholestyramine on endothelial function in patients with serum low-density lipoprotein cholesterol levels <160 mg/dl and high-density lipoprotein cholesterol levels <40 mg/dl. *Am. J. Cardiol.* 80 831–835. 10.1016/s0002-9149(97)00531-6 9381993

[B4] Bajwa A. Wesolowski R. Patel A. Saha P. Ludwinski F. Ikram M. (2016). Blood Oxygenation level-dependent CMR-derived measures in critical limb ischemia and changes with revascularization. *JACC* 67 420–431. 10.1016/j.jacc.2015.10.085 26821631 PMC4728170

[B5] Baker W. B. Li Z. Schenkel S. S. Chandra M. Busch D. R. Englund E. K. (2017). Effects of exercise training on calf muscle oxygen extraction and blood flow in patients with peripheral artery disease. *J. Appl. Physiol.* 123 1599–1609. 10.1152/japplphysiol.00585.2017 28982943 PMC5814687

[B6] Bakermans A. J. Wessel C. H. Zheng K. H. Groot P. F. C. Stroes E. S. G. Nederveen A. J. (2019). Dynamic magnetic resonance measurements of calf muscle oxygenation and energy metabolism in peripheral artery disease. *J. Magn. Reson. Imaging* 51 98–107. 10.1002/jmri.26841 31218803 PMC6916546

[B7] Bernstein E. F. Murphy A. E. Shea M. A. Housman L. B. (1970). Experimental and clinical experience with transcutaneous doppler ultrasonic flowmeters. *AMA Arch. Surg.* 101 21–25. 5419728 10.1001/archsurg.1970.01340250023006

[B8] Bhagat K. Hingorani A. Vallance P. (1997). Flow associated or flow mediated dilatation? More than just semantics. *Heart* 78 7–8. 10.1136/hrt.78.1.7 9290392 PMC484854

[B9] Boushel R. Piantadosi C. A. (2000). Near-infrared spectroscopy for monitoring muscle oxygenation. *Acta Physiol. Scand.* 168 615–622. 10.1046/j.1365-201x.2000.00713.x 10759598

[B10] Bryant D. J. Payne J. A. Firmin D. N. Longmore D. B. (1984). Measurement of flow with NMR imaging using a gradient pulse and phase difference technique. *J. Comput. Assist. Tomogr.* 8 588–593. 10.1097/00004728-198408000-00002 6736356

[B11] Buonocore M. H. (1998). Visualizing blood flow patterns using streamlines, arrows, and particle paths. *Magn. Reson. Med.* 40 210–226. 10.1002/mrm.1910400207 9702703

[B12] Buxton R. B. Frank L. R. Wong E. C. Siewert B. Warach S. Edelman R. R. (1998). A general kinetic model for quantitative perfusion imaging with arterial spin labeling. *Magn. Reson. Med.* 40 383–396. 10.1002/mrm.1910400308 9727941

[B13] Caporale A. Langham M. C. Guo W. Johncola A. Chatterjee S. Wehrli F. W. (2019). Acute effects of electronic cigarette aerosol inhalation on vascular function detected at quantitative MRI. *Radiology* 293 97–106. 10.1148/radiol.2019190562 31429679 PMC6776371

[B14] Carlsson I. Sollevi A. Wennmalm A. (1987). The role of myogenic relaxation, adenosine and prostaglandins in human forearm reactive hyperaemia. *J. Physiol.* 389 147–161. 10.1113/jphysiol.1987.sp016651 3681724 PMC1192075

[B15] Celermajer D. S. Sorensen K. E. Gooch V. M. Spiegelhalter D. J. Miller O. I. Sullivan I. D. (1992). Non-invasive detection of endothelial dysfunction in children and adults at risk of atherosclerosis. *Lancet* 340 1111–1115. 10.1016/0140-6736(92)93147-f 1359209

[B16] Chen H.-J. Wright G. A. (2017). A physiological model for interpretation of arterial spin labeling reactive hyperemia of calf muscles. *PLoS One* 12:e0183259. 10.1371/journal.pone.0183259 28837695 PMC5570335

[B17] Crecelius A. R. Richards J. C. Luckasen G. J. Larson D. G. Dinenno F. A. (2013). Reactive hyperemia occurs via activation of inwardly rectifying potassium channels and Na +/K +-ATPase in Humans. *Circ. Res.* 113 1023–1032. 10.1161/CIRCRESAHA.113.301675 23940309 PMC3871189

[B18] Damon B. M. Hornberger J. L. Wadington M. C. Lansdown D. A. Kent-Braun J. A. (2007). Dual gradient-echo MRI of post-contraction changes in skeletal muscle blood volume and oxygenation. *Magn. Reson. Med.* 57 670–679. 10.1002/mrm.21191 17390346 PMC4437703

[B19] Davignon J. (2004). Role of Endothelial Dysfunction In Atherosclerosis. *Circulation* 109 III–27–III–32. 10.1161/01.CIR.0000131515.03336.f8 15198963

[B20] de Roos N. M. Bots M. L. Katan M. B. (2001). Replacement of dietary saturated fatty acids by trans fatty acids lowers serum HDL cholesterol and impairs endothelial function in healthy men and women. *Atheroscler. Thromb. Vasc Biol.* 21 1233–1237. 10.1161/hq0701.092161 11451757

[B21] Detre J. A. Leigh J. S. Williams D. S. Koretsky A. P. (1992). Perfusion imaging. *Magn. Reson. Med.* 23 37–45. 10.1002/mrm.1910230106 1734182

[B22] Englund E. K. Langham M. C. Li C. Rodgers Z. B. Floyd T. F. Mohler E. R. (2013). Combined measurement of perfusion, venous oxygen saturation, and skeletal muscle T_2_^∗^ during reactive hyperemia in the leg. *J. Cardiovasc. Magn. Reson.* 15:70. 10.1186/1532-429X-15-70 23958293 PMC3765712

[B23] Englund E. K. Langham M. C. Ratcliffe S. J. Fanning M. J. Wehrli F. W. Mohler E. R. (2015). Multiparametric assessment of vascular function in peripheral artery disease: dynamic measurement of skeletal muscle perfusion, blood-oxygen-level dependent signal, and venous oxygen saturation. *Circ.Cardiovasc. Imaging* 8:e002673. 10.1161/CIRCIMAGING.114.002673 25873722 PMC4399002

[B24] Englund E. K. Rodgers Z. B. Langham M. C. Mohler E. R. III Floyd T. F. Wehrli F. W. (2016). Measurement of skeletal muscle perfusion dynamics with pseudo-continuous arterial spin labeling (pCASL): assessment of relative labeling efficiency at rest and during hyperemia, and comparison to pulsed arterial spin labeling (PASL). *J. Magn. Reson. Imaging* 44 929–939. 10.1002/jmri.25247 27043039 PMC5424607

[B25] Englund E. K. Rodgers Z. B. Langham M. C. Mohler E. R. III Floyd T. F. Wehrli F. W. (2017). Simultaneous measurement of macro- and microvascular blood flow and oxygen saturation for quantification of muscle oxygen consumption. *Magn. Reson. Med.* 79 846–855. 10.1002/mrm.26744 28497497 PMC5681899

[B26] Fernández-Seara M. A. Techawiboonwong A. Detre J. A. Wehrli F. W. (2006). MR susceptometry for measuring global brain oxygen extraction. *Magn. Reson. Med.* 55 967–973. 10.1002/mrm.20892 16598726

[B27] Fisher J. A. Venkatraghavan L. Mikulis D. J. (2018). Magnetic resonance imaging-based cerebrovascular reactivity and hemodynamic reserve. *Stroke* 49 2011–2018. 10.1161/STROKEAHA.118.021012 29986929

[B28] Flammer A. J. Anderson T. Celermajer D. S. Creager M. A. Deanfield J. Ganz P. (2012). The assessment of endothelial function: from research into clinical practice. *Circulation* 126 753–767. 10.1161/CIRCULATIONAHA.112.093245 22869857 PMC3427943

[B29] Fronek A. Johansen K. Dilley R. B. Bernstein E. F. (1973). Ultrasonographically monitored postocclusive reactive hyperemia in diagnosis of peripheral arterial occlusive disease. *Circulation* 48 149–152. 10.1161/01.cir.48.1.149 4781233

[B30] Frydrychowicz A. Winterer J. T. Zaitsev M. Jung B. Hennig J. Langer M. (2007). Visualization of iliac and proximal femoral artery hemodynamics using time-resolved 3D phase contrast MRI at 3T. *J. Magn. Reson. Imaging* 25 1085–1092. 10.1002/jmri.20900 17427916

[B31] Furchgott R. F. Zawadzki J. V. (1980). The obligatory role of endothelial-cells in the relaxation of arterial smooth-muscle by acetylcholine. *Nature* 288 373–376. 10.1038/288373a0 6253831

[B32] Glover G. H. (2011). Overview of functional magnetic resonance imaging. *Neurosurg. Clin. N. Am.* 22 133–139. 10.1016/j.nec.2010.11.001 21435566 PMC3073717

[B33] Grözinger G. Pohmann R. Schick F. Grosse U. Syha R. Brechtel K. (2013). Perfusion measurements of the calf in patients with peripheral arterial occlusive disease before and after percutaneous transluminal angioplasty using Mr arterial spin labeling. *J. Magn. Reson. Imaging* 40 980–987. 10.1002/jmri.24463 24243496

[B34] Haacke E. M. Lai S. Reichenbach J. R. Kuppusamy K. Hoogenraad F. Takeichi H. (1997). In vivo measurement of blood oxygen saturation using magnetic resonance imaging: a direct validation of the blood oxygen level-dependent concept in functional brain imaging. *Hum Brain Mapp* 5 341–346. 10.1002/(SICI)1097-019319975:5 20408238

[B35] Hardie K. L. Kinlay S. Hardy D. B. Wlodarczyk J. Silberberg J. S. Fletcher P. J. (1997). Reproducibility of brachial ultrasonography and flow-mediated dilatation (FMD) for assessing endothelial function. *Aust. N. Z. J. Med.* 27 649–652. 10.1111/j.1445-5994.1997.tb00992.x 9483230

[B36] Harrison D. G. Freiman P. C. Armstrong M. L. Marcus M. L. Heistad D. D. (1987). Alterations of vascular reactivity in atherosclerosis. *Circ. Res.* 61 II74–II80. 2822287

[B37] He X. Yablonskiy D. A. (2006). Quantitative BOLD: mapping of human cerebral deoxygenated blood volume and oxygen extraction fraction: default state. *Magn. Reson. Med.* 57 115–126. 10.1002/mrm.21108 17191227 PMC3971521

[B38] Heinonen I. Kemppainen J. Kaskinoro K. Peltonen J. E. Borra R. Lindroos M. M. (2010). Comparison of exogenous adenosine and voluntary exercise on human skeletal muscle perfusion and perfusion heterogeneity. *J. Appl. Physiol.* 108 378–386. 10.1152/japplphysiol.00745.2009 19940098

[B39] Huegli R. W. Schulte A.-C. Aschwanden M. Thalhammer C. Kos S. Jacob A. L. (2008). Effects of percutaneous transluminal angioplasty on muscle BOLD-MRI in patients with peripheral arterial occlusive disease: preliminary results. *Eur. Radiol.* 19 509–515. 10.1007/s00330-008-1168-6 18795296

[B40] Isbell D. C. Epstein F. H. Zhong X. DiMaria J. M. Berr S. S. Meyer C. H. (2007). Calf muscle perfusion at peak exercise in peripheral arterial disease: measurement by first-pass contrast-enhanced magnetic resonance imaging. *J. Magn. Reson. Imaging* 25 1013–1020. 10.1002/jmri.20899 17410566 PMC2930771

[B41] Kim S. G. (1995). Quantification of relative cerebral blood flow change by flow-sensitive alternating inversion recovery (FAIR) technique: application to functional mapping. *Magn. Reson. Med.* 34 293–301. 10.1002/mrm.1910340303 7500865

[B42] Kim S.-G. Ogawa S. (2012). Biophysical and physiological origins of blood oxygenation level-dependent fMRI signals. *JCBFM* 32 1188–1206. 10.1038/jcbfm.2012.23 22395207 PMC3390806

[B43] Kim S. G. Tsekos N. V. Berr S. S. (1997). Perfusion imaging by a flow-sensitive alternating inversion recovery (FAIR) technique: application to functional brain imaging. *Magn. Reson. Med.* 37 425–435. 10.1002/mrm.1910370321 9055234

[B44] Kos S. Klarhöfer M. Aschwanden M. (2009). Simultaneous dynamic blood oxygen level-dependent magnetic resonance imaging of foot and calf muscles: aging effects at ischemia and postocclusive hyperemia in …. *Invest. Radiol.* 44 741–747. 10.1097/rli.0b013e3181b248f9 19809343

[B45] Lakatta E. G. Levy D. (2003). Arterial and cardiac aging: major shareholders in cardiovascular disease enterprises. *Circulation* 107 139–146. 10.1161/01.CIR.0000048892.83521.58 12515756

[B46] Langham M. C. Desjardins B. Englund E. K. Mohler E. R. III Floyd T. F. Wehrli F. W. (2016). Rapid high-resolution, self-registered, dual lumen-contrast MRI method for vessel-wall assessment in peripheral artery disease. *Acad. Radiol.* 23 457–467. 10.1016/j.acra.2015.12.015 26916248 PMC5654744

[B47] Langham M. C. Englund E. K. Mohler E. R. III Li C. Rodgers Z. B. Floyd T. F. (2013a). Quantitative CMR markers of impaired vascular reactivity associated with age and peripheral artery disease. *J. Cardiovasc. Magn. Reson.* 15 1–10. 10.1186/1532-429X-15-17 23402422 PMC3599649

[B48] Langham M. C. Floyd T. F. Mohler E. R. Magland J. F. Wehrli F. W. (2010a). Evaluation of cuff-induced ischemia in the lower extremity by magnetic resonance oximetry. *JACC* 55 598–606. 10.1016/j.jacc.2009.08.068 20152564 PMC2833093

[B49] Langham M. C. Jain V. Magland J. F. Wehrli F. W. (2010b). Time-resolved absolute velocity quantification with projections. *Magn. Reson. Med.* 64 1599–1606. 10.1002/mrm.22559 20677235 PMC2974053

[B50] Langham M. C. Li C. Englund E. K. Chirico E. N. Mohler E. R. Floyd T. F. (2013b). Vessel-wall imaging and quantification of flow-mediated dilation using water-selective 3D SSFP-echo. *J. Cardiovasc. Magn. Reson.* 15 1–9. 10.1186/1532-429X-15-100 24172037 PMC3819508

[B51] Langham M. C. Li C. Wehrli F. W. (2011). Non-triggered quantification of central and peripheral pulse-wave velocity. *J. Cardiovasc. Magn. Reson.* 13:81. 10.1186/1532-429X-13-81 22188972 PMC3258212

[B52] Langham M. C. Wehrli F. W. (2011). Simultaneous mapping of temporally-resolved blood flow velocity and oxygenation in femoral artery and vein during reactive hyperemia. *J. Cardiovasc. Magn. Reson.* 13 66–68. 10.1186/1532-429X-13-66 22035402 PMC3223132

[B53] Langham M. C. Zhou Y. Chirico E. N. Magland J. F. Sehgal C. M. Englund E. K. (2015). Effects of age and smoking on endothelial function assessed by quantitative cardiovascular magnetic resonance in the peripheral and central vasculature. *J. Cardiovasc. Magn. Reson.* 17:19. 10.1186/s12968-015-0110-8 25884943 PMC4332734

[B54] Larsen R. G. Thomsen J. M. Hirata R. P. Steffensen R. Poulsen E. R. Frøkjær J. B. (2019). Impaired microvascular reactivity after eccentric muscle contractions is not restored by acute ingestion of antioxidants or dietary nitrate. *Physiol. Rep.* 7 1102–1115. 10.14814/phy2.14162 31293100 PMC6640596

[B55] Lebon V. Brillault-Salvat C. Bloch G. Leroy-Willig A. Carlier P. G. (1998). Evidence of muscle BOLD effect revealed by simultaneous interleaved gradient-echo NMRI and myoglobin NMRS during leg ischemia. *Magn. Reson. Med.* 40 551–558. 10.1002/mrm.1910400408 9771572

[B56] Ledermann H. P. Schulte A. C. Heidecker H. G. Aschwanden M. Jager K. A. Scheffler K. (2006). Blood oxygenation level-dependent magnetic resonance imaging of the skeletal muscle in patients with peripheral arterial occlusive disease. *Circulation* 113 2929–2935. 10.1161/CIRCULATIONAHA.105.605717 16785340

[B57] Li Z. Muller M. D. Wang J. Sica C. T. Karunanayaka P. Sinoway L. I. (2016). Dynamic characteristics of T2^∗^-weighted signal in calf muscles of peripheral artery disease during low-intensity exercise. *J. Magn. Reson. Imaging* 46 40–48. 10.1002/jmri.25532 27783446 PMC5406273

[B58] Lieberman E. H. Gerhard M. D. Uehata A. Selwyn A. P. Ganz P. Yeung A. C. (1996). Flow-induced vasodilation of the human brachial artery is impaired in patients <40 years of age with coronary artery disease. *Am. J. Cardiol.* 78 1210–1214. 10.1016/s0002-9149(96)00597-8 8960576

[B59] Lu H. Ge Y. (2008). Quantitative evaluation of oxygenation in venous vessels using T2-Relaxation-Under-Spin-Tagging MRI. *Magn. Reson. Med.* 60 357–363. 10.1002/mrm.21627 18666116 PMC2587050

[B60] Markl M. Frydrychowicz A. Kozerke S. Hope M. Wieben O. (2012). 4D flow MRI. *J. Magn. Reson. Imaging* 36 1015–1036. 10.1002/jmri.23632 23090914

[B61] Mathewson K. W. Haykowsky M. J. Thompson R. B. (2014). Feasibility and reproducibility of measurement of whole muscle blood flow, oxygen extraction, and VO 2with dynamic exercise using MRI. *Magn. Reson. Med.* 74 1640–1651. 10.1002/mrm.25564 25533515

[B62] Messner B. Bernhard D. (2014). Smoking and cardiovascular disease. *Atheroscler. Thromb. Vasc Biol.* 34 509–515. 10.1161/ATVBAHA.113.300156 24554606

[B63] Mohajer K. Zhang H. Gurell D. Ersoy H. Ho B. Kent K. C. (2006). Superficial femoral artery occlusive disease severity correlates with MR cine phase-contrast flow measurements. *J. Magn. Reson. Imaging* 23 355–360. 10.1002/jmri.20514 16463304

[B64] Mohiaddin R. H. Gatehouse E. D. Moon J. C. C. Youssuffidin M. Yang G. Z. Firmin D. N. (2002). Assessment of reactive hyperaemia using real time zonal echo-planar flow imaging. *J. Cardiovasc. Magn. Reson.* 4 283–287. 10.1081/jcmr-120003954 12074143

[B65] Moran P. R. (1982). A flow velocity zeugmatographic interlace for NMR imaging in humans. *Magn. Reson. Imaging* 1 197–203. 10.1016/0730-725x(82)90170-9 6927206

[B66] Moran P. R. Moran R. A. Karstaedt N. (1985). Verification and evaluation of internal flow and motion. True magnetic resonance imaging by the phase gradient modulation method. *Radiology* 154 433–441. 10.1148/radiology.154.2.3966130 3966130

[B67] Murphy T. P. Cutlip D. E. Regensteiner J. G. Mohler E. R. Cohen D. J. Reynolds M. R. (2012). Supervised exercise versus primary stenting for claudication resulting from aortoiliac peripheral artery disease. *Circulation* 125 130–139. 10.1161/CIRCULATIONAHA.111.075770 22090168 PMC3374869

[B68] Nayak K. S. Nielsen J.-F. Bernstein M. A. Markl M. Gatehouse P. Botnar R. (2015). Cardiovascular magnetic resonance phase contrast imaging. *J. Cardiovasc. Magn. Reson.* 17:71. 10.1186/s12968-015-0172-7 26254979 PMC4529988

[B69] Nayler G. L. Firmin D. N. Longmore D. B. (1986). Blood flow imaging by cine magnetic resonance. *J. Comput. Assist. Tomogr.* 10 715–722. 3528245 10.1097/00004728-198609000-00001

[B70] Nioka S. Kime R. Sunar U. Im J. Izzetoglu M. Zhang J. (2006). A novel method to measure regional muscle blood flow continuously using NIRS kinetics information. *Dyn. Med.* 5:5. 10.1186/1476-5918-5-5 16704736 PMC1540409

[B71] Nishii T. Kono A. K. Nishio M. Kyotani K. Nishiyama K. Sugimura K. (2015). Dynamic blood oxygen level-dependent MR imaging of muscle: comparison of postocclusive reactive hyperemia in young smokers and nonsmokers. *MRMS* 14 275–283. 10.2463/mrms.2014-0105 25994035

[B72] Partovi S. Aschwanden M. Jacobi B. Schulte A.-C. Walker U. A. Staub D. (2013). Correlation of muscle BOLD MRI with transcutaneous oxygen pressure for assessing microcirculation in patients with systemic sclerosis. *J. Magn. Reson. Imaging* 38 845–851. 10.1002/jmri.24046 23441019

[B73] Partovi S. Karimi S. Jacobi B. Schulte A.-C. Aschwanden M. Zipp L. (2012a). Clinical implications of skeletal muscle blood-oxygenation-level-dependent (BOLD) MRI. *Magn. Reson. Mater. Phy.* 25 251–261. 10.1007/s10334-012-0306-y 22374263

[B74] Partovi S. Schulte A.-C. Aschwanden M. Staub D. Benz D. Imfeld S. (2012b). Impaired skeletal muscle microcirculation in systemic sclerosis. *Arthritis Res. Ther.* 14:R209. 10.1186/ar4047 23036642 PMC3580521

[B75] Pauling L. Coryell C. D. (1936). The magnetic properties and structure of hemoglobin, oxyhemoglobin and carbonmonoxyhemoglobin. *PNAS* 22 210–216. 10.1073/pnas.22.4.210 16577697 PMC1076743

[B76] Potter K. Reed C. J. Green D. J. Hankey G. J. Arnolda L. F. (2008). Ultrasound settings significantly alter arterial lumen and wall thickness measurements. *Cardiovasc Ultrasound* 6 10.1186/1476-7120-6-6 18208623 PMC2254584

[B77] Pyke K. E. Tschakovsky M. E. (2005). The relationship between shear stress and flow-mediated dilatation: implications for the assessment of endothelial function. *J. Physiol.* 568 357–369. 10.1113/jphysiol.2005.089755 16051630 PMC1474741

[B78] Raichle M. E. (1998). Behind the scenes of functional brain imaging: a historical and physiological perspective. *PNAS* 95 765–772. 10.1073/pnas.95.3.765 9448239 PMC33796

[B79] Raynaud J. S. Duteil S. Vaughan J. T. Hennel F. Wary C. Leroy-Willig A. (2001). Determination of skeletal muscle perfusion using arterial spin labeling NMRI: validation by comparison with venous occlusion plethysmography. *Magn. Reson. Med.* 46 1–7. 10.1002/mrm.1192 11477634

[B80] Richards J. C. Racine M. L. Hearon C. M. Jr. Kunkel M. Luckasen G. J. Larson D. G. (2018). Acute ingestion of dietary nitrate increases muscle blood flow via local vasodilation during handgrip exercise in young adults. *Physiol. Rep.* 6:e13572 10.14814/phy2.13572 29380952 PMC5789727

[B81] Sanchez O. A. Copenhaver E. A. Elder C. P. Damon B. M. (2010). Absence of a significant extravascular contribution to the skeletal muscle BOLD effect at 3 T. *Magn. Reson. Med.* 64 527–535. 10.1002/mrm.22449 20665796 PMC2914541

[B82] Sanne H. Sivertsson R. (1968). The effect of exercise on the development of collateral circulation after experimental occlusion of the femoral artery in the cat. *Acta Physiol. Scand.* 73 257–263. 10.1111/j.1748-1716.1968.tb04104.x 5709583

[B83] Schulte A.-C. Aschwanden M. Bilecen D. (2008). Calf muscles at blood oxygen level–dependent MR imaging: aging effects at postocclusive reactive hyperemia. *Radiology* 247 482–489. 10.1148/radiol.2472070828 18372453

[B84] Sejda T. Pit’ha J. Svandova E. Poledne R. (2005). Limitations of non-invasive endothelial function assessment by brachial artery flow-mediated dilatation. *Clin. Physiol. Funct. Imaging* 25 58–61. 10.1111/j.1475-097X.2004.00590.x 15659082

[B85] Silber H. A. Bluemke D. A. Ouyang P. Du Y. P. Post W. S. Lima J. A. C. (2001). The relationship between vascular wall shear stress and flow-mediated dilation: endothelial function assessed by phase-contrast magnetic resonance angiography. *JACC* 38 1859–1865. 10.1016/S0735-1097(01)01649-7 11738285

[B86] Sorensen K. E. Celermajer D. S. Spiegelhalter D. J. Georgakopoulos D. Robinson J. Thomas O. (1995). Non-invasive measurement of human endothelium dependent arterial responses: accuracy and reproducibility. *Br. Heart J.* 74 247–253. 10.1136/hrt.74.3.247 7547018 PMC484014

[B87] Sorensen M. B. Collins P. Ong P. J. L. Webb C. M. Hayward C. S. Asbury E. A. (2002). Long-term use of contraceptive depot medroxyprogesterone acetate in young women impairs arterial endothelial function assessed by cardiovascular magnetic resonance. *Circulation* 106 1646–1651. 10.1161/01.CIR.0000030940.73167.4E 12270857

[B88] Tagawa T. Imaizumi T. Endo T. Shiramoto M. Harasawa Y. Takeshita A. (1994). Role of nitric oxide in reactive hyperemia in human forearm vessels. *Circulation* 90 2285–2290. 10.1161/01.cir.90.5.2285 7955185

[B89] Tonson A. Noble K. E. Meyer R. A. Rozman M. R. Foley K. T. Slade J. M. (2017). Age reduces microvascular function in the leg independent of physical activity. *Med. Sci. Sports Exerc.* 49 1623–1630. 10.1249/MSS.0000000000001281 28709153 PMC5561656

[B90] Towse T. F. Elder C. P. Bush E. C. Klockenkemper S. W. Bullock J. T. Dortch R. D. (2016). Post-contractile BOLD contrast in skeletal muscle at 7 T reveals inter-individual heterogeneity in the physiological responses to muscle contraction. *NMR Biomed.* 29 1720–1728. 10.1002/nbm.3593 27753155 PMC6594689

[B91] Versluis B. Dremmen M. H. G. Nelemans P. J. Wildberger J. E. Schurink G.-W. Leiner T. (2012). MRI of arterial flow reserve in patients with intermittent claudication: feasibility and initial experience. *PLoS One* 7:e31514. 10.1371/journal.pone.0031514 22412836 PMC3297594

[B92] Versluis B. Nelemans P. J. Wildberger J. E. Schurink G. W. Leiner T. Backes W. H. (2014). Magnetic resonance imaging-derived arterial peak flow in peripheral arterial disease: towards a standardized measurement. *Eur. J. Vasc Endovasc. Surg.* 48 185–192. 10.1016/j.ejvs.2014.04.022 24923235

[B93] Wentland A. L. Grist T. M. Wieben O. (2014). Review of MRI-based measurements of pulse wave velocity: a biomarker of arterial stiffness. *Cardiovasc. Diagn. Ther.* 4 193–206. 10.3978/j.issn.2223-3652.2014.03.04 24834415 PMC3996237

[B94] Widlansky M. E. Gokce N. Keaney J. F. Jr. Vita J. A. (2003). The clinical implications of endothelial dysfunction. *JACC* 42 1149–1160. 10.1016/S0735-1097(03)00994-X 14522472

[B95] Wiesmann F. Petersen S. E. Leeson P. M. Francis J. M. Robson M. D. Wang Q. (2004). Global impairment of brachial, carotid, and aortic vascular function in young smokers. *JACC* 44 2056–2064. 10.1016/j.jacc.2004.08.033 15542292

[B96] Williams D. S. Detre J. A. Leigh J. S. Koretsky A. P. (1992). Magnetic resonance imaging of perfusion using spin inversion of arterial water. *PNAS* 89 212–216. 1729691 10.1073/pnas.89.1.212 PMC48206

[B97] Wong E. C. Buxton R. B. Frank L. R. (1997). Implementation of quantitative perfusion imaging techniques for functional brain mapping using pulsed arterial spin labeling. *NMR Biomed.* 10 237–249. 10.1002/(sici)1099-1492(199706/08)10:4/5<237::aid-nbm475>3.0.co;2-x 9430354

[B98] Wu W.-C. Mohler E. Ratcliffe S. J. Wehrli F. W. Detre J. A. Floyd T. F. (2009). Skeletal muscle microvascular flow in progressive peripheral artery disease: assessment with continuous arterial spin-labeling perfusion magnetic resonance imaging. *JACC* 53 2372–2377. 10.1016/j.jacc.2009.03.033 19539149 PMC2763280

[B99] Wu W. C. Wang J. Detre J. A. Wehrli F. W. Mohler E. Ratcliffe S. J. (2008). Hyperemic flow heterogeneity within the calf, foot, and forearm measured with continuous arterial spin labeling MRI. *AJP Heart Circ. Physiol.* 294 H2129–H2136. 10.1152/ajpheart.01399.2007 18310508 PMC3020668

[B100] Yamauchi T. Ohnaka K. Takayanagi R. Umeda F. Nawata H. (1990). Enhanced secretion of endothelin-1 by elevated glucose levels from cultured bovine aortic endothelial cells. *FEBS Lett.* 267 16–18. 10.1016/0014-5793(90)80276-o 2114322

[B101] Zeiher A. M. Drexler H. Saurbier B. Just H. (1993). Endothelium-mediated coronary blood flow modulation in humans. Effects of age, atherosclerosis, hypercholesterolemia, and hypertension. *J. Clin. Invest.* 92 652–662. 10.1172/JCI116634 8349804 PMC294898

[B102] Zhang J. L. Layec G. Hanrahan C. Conlin C. C. Hart C. Hu N. (2019). Exercise-induced calf muscle hyperemia: quantitative mapping with low-dose dynamic contrast enhanced magnetic resonance imaging. *AJP Heart Circ. Physiol.* 316 H201–H211. 10.1152/ajpheart.00537.2018 30388024 PMC6415822

[B103] Zheng J. An H. Coggan A. R. Zhang X. Bashir A. Muccigrosso D. (2013). Noncontrast skeletal muscle oximetry. *Magn. Reson. Med.* 71 318–325. 10.1002/mrm.24669 23424006 PMC3661680

[B104] Zheng J. Hastings M. K. Muccigross D. Fan Z. Gao F. Curci J. (2014). Non-contrast MRI perfusion angiosome in diabetic feet. *Eur. Radiol.* 25 99–105. 10.1007/s00330-014-3337-0 25100334

